# Spatial displacement of forward-diffracted X-ray beams by perfect crystals

**DOI:** 10.1107/S2053273318001419

**Published:** 2018-02-23

**Authors:** A. Rodriguez-Fernandez, V. Esposito, D. F. Sanchez, K. D. Finkelstein, P. Juranic, U. Staub, D. Grolimund, S. Reiche, B. Pedrini

**Affiliations:** a Paul Scherrer Institut, Villigen, Switzerland; b Cornell High Energy Synchrotron Source, Ithaca, NY, USA

**Keywords:** X-ray dynamical diffraction, perfect crystals, transverse echo displacement, hard X-ray self-seeding

## Abstract

Time-delayed, narrow-band echoes generated by forward Bragg diffraction of an X-ray pulse by a perfect thin crystal are exploited for self-seeding at hard X-ray free-electron lasers. Theoretical predictions indicate that the retardation is strictly correlated to a transverse displacement of the echo pulses. This article reports the first experimental observation of the displaced echoes. The displacements are in good agreement with simulations relying on the dynamical diffraction theory. The echo signals are characteristic for a given Bragg reflection, the structure factor and the probed interplane distance. The reported results pave the way to exploiting the signals as an online diagnostic tool for hard X-ray free-electron laser seeding and for dynamical diffraction investigations of strain at the femtosecond timescale.

## Abstract 2

The first experimental observation of transverse spatial echoes generated by forward Bragg diffraction of an X-ray beam propagating through a perfect thin crystal is reported.

## 1. Introduction   

Hard X-ray free-electron lasers (XFELs) are novel photon sources, which rely on the self-amplified spontaneous emission (SASE) process to obtain peak brightnesses in the soft and hard X-ray regime that are orders of magnitude larger than those achieved with insertion devices at third-generation synchrotron light sources (Margaritondo & Rebernik Ribic, 2011 ▸). The ultrashort pulse length opens new avenues for investigations of phenomena at the femtosecond timescale. The SASE radiation arises from amplification of stochastic noise in the electron bunch. Therefore, it consists of many longitudinal modes (Wark & Lee, 1999 ▸), and exhibits strong shot-to-shot fluctuations of both the mean pulse energy and the pulse spectrum. Furthermore, the relative bandwidth at an XFEL operating in SASE mode is typically of the order of 10^−3^. Many XFEL experiments require a much narrower bandwidth and excellent spectral stability (Alonso-Mori *et al.*, 2015 ▸). These beam properties can be enforced by inserting a monochromator in the X-ray beam path, at the expense of losing a large fraction of the beam intensity.

Self-seeding has been proposed as an intensity-efficient mode of operation for XFELs (Saldin *et al.*, 2001 ▸). After SASE amplification in a first undulator section, the electron bunch is separated from the photons and delayed by a magnetic chicane, which also refreshes the electron bunch by suppressing the microbunching that results from the SASE process. The X-ray photon pulse is unaffected by the chicane and propagates straight to a monochromatizing optical element that delays a narrow-bandwidth pulse from the main SASE pulse, while the rest of the energies of the SASE pulse propagate unperturbed. Hence, the electron-bunch retardation is essential for the longitudinal overlap of the electron bunch with the retarded photon pulse, which acts as a narrow-band seed for the FEL amplification along the second undulator section. The XFEL pulse thus obtained is characterized by the same narrow bandwidth and by a stable wavelength set by the monochromator.

In the hard X-ray regime, monochromators are typically based on perfect crystals. Geloni *et al.* (2010 ▸) have proposed generating the narrow-band seed with a thin crystal in the Bragg condition. This process of forward Bragg diffraction (FBD) is described by the dynamical diffraction theory (Zachariasen, 1945 ▸; Batterman & Cole, 1964 ▸; Shvydko, 2004 ▸), which accounts for multiple-scattering effects relevant in perfect crystals. For most of the radiation in the incoming SASE pulse the crystal is transparent. Only the wavelengths that are close to or satisfy the Bragg condition are affected, and a series of time-delayed pulses of low intensity but narrow bandwidth, called echoes, are generated in the temporal tail of the transmitted pulse (Geloni *et al.*, 2010 ▸; Shvydko & Lindberg, 2012 ▸; Yamg & Shvydko, 2013 ▸). This self-seeding scheme has been demonstrated experimentally at the Linac Coherent Light Source (LCLS) XFEL. The relevant publication (Amann *et al.*, 2012 ▸) hints at the fact that the echoes used to seed the electron pulse in the downstream undulator section are subject to a transverse displacement, a phenomenon predicted to be closely related to the retardation of the echoes (Shvydko & Lindberg, 2012 ▸).

There is great interest in the fine-tuning possibilities of the FBD process, mainly in view of the possible future implementation of self-seeding at *e.g.* the hard X-ray beamline ARAMIS of the Swiss X-ray free-electron laser (SwissFEL) (Milne *et al.*, 2017 ▸). The work presented here aims to gain a better understanding of space-, time- and frequency-domain effects in the FBD process. The results reported represent the first direct and unambiguous experimental evidence of the spatially displaced echoes in the forward transmitted photon beam, made possible using an X-ray beam focused down to the micrometre scale. Our results are backed up by simulations that confirm the interpretation of the experimental signals in terms of FBD echoes.

The article is structured as follows. In §2 of this work, the key points of the dynamical diffraction theory relevant to the FBD problem are reviewed and their implementation in the simulation tools is described. §3 describes the diffraction experiments. The results are reported in §4, which include the comparison with the outcome of the simulations. In §5 the results are discussed from a wider perspective and the possible applications are addressed.

## 2. Theory   

### 2.1. Dynamical diffraction effects on propagation of X-rays through a perfect crystal   

**Figure 1:**
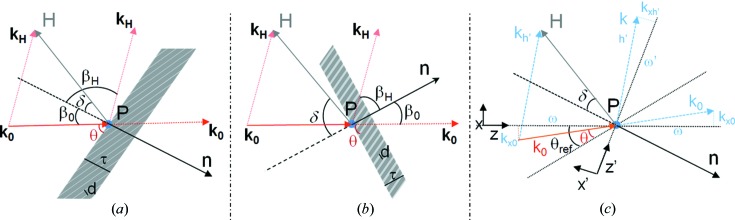
Sketch of the geometry of diffraction from a thin crystal in (*a*) Bragg geometry and (*b*) Laue geometry. The crystal of thickness τ, with the Bragg vector 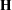 and the surface normal 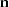, is represented in grey with the white stripes parallel to the Bragg diffraction planes. The wavevector of the incident and transmitted beam is 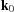, while that for the diffracted beam is 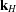. The incident angle θ, the asymmetry angle δ and the angle differences 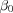 and 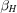 are indicated. *P* is a reference point on the crystal entrance surface. (*c*) Illustration of the coordinate system 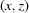 for the incoming and transmitted beam, and 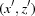 for the diffracted beam. 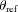 is the incidence angle for the *z* direction.

Consider the situation represented in Fig. 1 ▸, in which an incoming X-ray beam hits a perfect thin crystal of infinite transverse extension. If the crystal is oriented such that the Bragg condition for a certain reflection is satisfied, part of the X-ray intensity is diffracted. The precise redistribution between transmitted and diffracted intensity is subtle, especially for thin crystals, and is intimately related to the echo phenomenon under consideration.

First, an incident plane wave is considered (Fig. 1 ▸). The incident wavevector 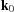, the Bragg vector 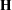 of the considered crystal reflection and the surface unit normal 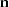 are restricted to lie in the same plane, corresponding to the drawing plane of the figure. The length *k* of the wavevector is related to the photon wavelength λ by 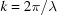 and the corresponding photon energy is 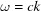 with *c* the speed of light. The length of the Bragg vector is related to the diffraction plane separation *d* by 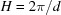. The incidence angle θ is the angle between 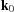 and 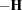, and the asymmetry parameter δ is the angle between 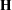 and 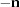. The Bragg condition is 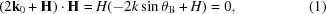
which sets the Bragg angle 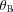 for a given photon energy. The wavevector of the transmitted plane wave is the same as that of the incident wave. The wavevector 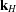 of the diffracted wave has the same length *k* and its direction is uniquely defined by requiring that the difference vector 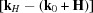 is parallel to 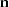 (Batterman & Cole, 1964 ▸). We denote 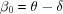, 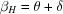 and 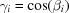, with 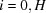. Other parameters of the crystal that are relevant for the diffraction process are the crystal thickness τ, the unit-cell volume *V*, and the Fourier transforms of the unit-cell structure factors 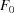, 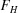 and 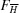 at momentum transfer zero, 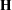 and 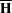.

The diffraction process is described by the transmission and reflection coefficients *R* and *T*. The meaning of these coefficients is that an incident plane wave 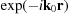 leads to a transmitted plane wave 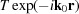 plus a diffracted wave 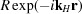. The coefficients are obtained by imposing suitable boundary conditions for the electric and magnetic fields at the two crystal surfaces between the Maxwell equation solutions outside the crystal, which are plane waves, and the solutions of the Maxwell equations inside the crystal derived from the dynamical diffraction theory (Zachariasen, 1945 ▸; Batterman & Cole, 1964 ▸). For Bragg geometry (Fig. 1 ▸ *a*), in which the diffracted wave is exiting by the same crystal surface as the incoming wave (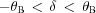), the formulas are
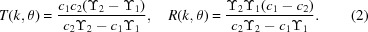
For the opposite Laue geometry, in which the diffracted wave is exiting by the same crystal surface as the transmitted wave (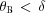), the formulas are
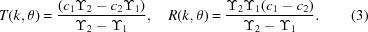
The expressions appearing in the above formulas are 
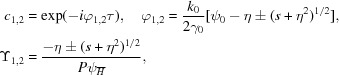
whereby 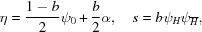
with 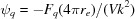 (where *r_e_* is the classical electron radius) for 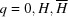. The basic parameters *b* and α are 
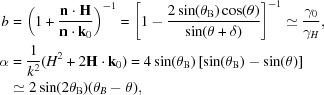
whereby the last approximations are valid for small differences 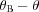.

Next, X-ray beams of finite transverse extension and small divergence are considered, and are described in the Fourier space approach (Fig. 1 ▸ *c*). To this end, for each beam a spatial coordinate system 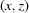 has to be fixed, with *z* the coordinate in the direction of propagation and *x* the transverse coordinate. A beam is then defined by specifying the Fourier components of the electric field 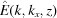 at any longitudinal point *z*, from which the field on the corresponding transverse plane and as a function of time is obtained by Fourier transformation: 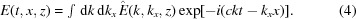
The superposition of photon energies *ck* and of transverse wavevector components 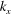 gives a time and a spatial structure, respectively. The components of the same beam at two different longitudinal coordinates 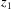 and 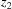 are related by the free-space propagation 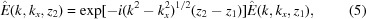
which is a simple phase multiplication. One recognizes that the Fourier component 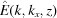 is associated with a plane wave with wavevector 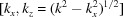, forming the small angle 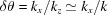 with the *z* direction.

The coordinate system 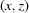 shown in Fig. 1 ▸(*c*) is suitable for both the incident and the transmitted beam. The *z* direction can be set to a reference incidence angle 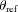 corresponding to the direction of the incident beam. Similarly, the coordinate system 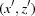 shown in the figure can be set with 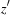 in the direction of the diffracted beam.

Let us denote by 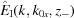 the Fourier components of the incident X-ray beam hitting the crystal at the spatial point *P* as shown in Fig. 1 ▸(*c*), with 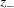 the longitudinal coordinate directly upstream of the crystal in the 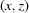 coordinate system. The transmission and reflection coefficients above are now used to propagate the beam through the thin crystals. The Fourier components of the transmitted beam at the coordinate 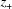 right after the crystal are indeed given by 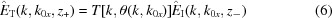
where the diffraction angle associated with the wavevector 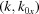 is given by 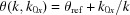. The Fourier components of the diffracted beam at the coordinate 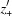 right after point *P* are given by the propagation 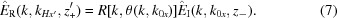
In this equation, 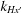 is the transverse wavevector component of the diffracted wavevector 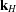, and it depends on *k* and 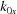.

**Figure 2:**
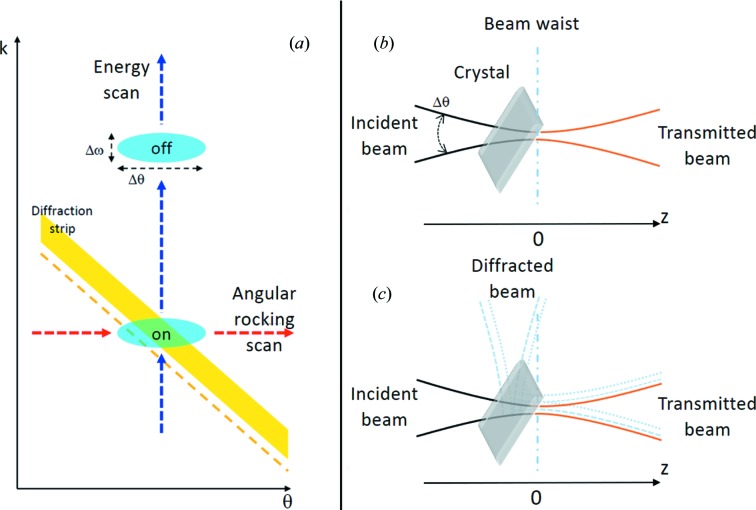
(*a*) DuMond diagram with sketched Bragg-condition line (dashed orange line) and diffraction region (yellow strip). The two cyan ovals are examples of an off- and an on-diffraction condition beam, with bandwidth 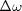 and divergence 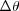. The dashed red and blue lines represent examples of angular and energy scans, respectively. The sketch is almost to scale for the (220) diamond reflection in the Bragg condition at 12 keV (see text). (*b*), (*c*) Sketches of the crystals, the X-ray beams and X-ray pulses for the off- and on-diffraction conditions, respectively. The converging incident beam comes from the right, has a waist at 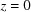 and hits the crystal upstream of the waist. In the off-diffraction case (*b*), the diverging transmitted beam matches the free-space propagation of the incoming beam after accounting for overall absorption. In the on-diffraction case (*c*), part of the intensity is redirected to the diffracted beam. Both transmitted and diffracted beams exhibit a substructure in the lateral spatial dimension.

The detailed features of the transmitted and diffracted beams are encoded in the form of *T* and *R*. Fig. 2 ▸ provides a qualitative illustration. Panel (*a*) is a DuMond diagram (DuMond, 1937 ▸), with axes *k* and 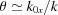, in which the incoming beam is represented as an oval. The dashed line corresponds to Bragg’s law for the reflection *d*-spacing, while the yellow strip corresponds to the Darwin acceptance of the reflection taking account of refraction at the surface, in which the reflection coefficient 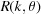 is significantly different from zero. Its precise position depends on the asymmetry of the diffraction geometry. Examples of the incoming beam are sketched as ovals for the off- and on-diffraction conditions. In the former case, the transmitted beam matches the incoming beam upon overall attenuation because of absorption effects in the crystal, and the intensity of the diffracted beam vanishes (Fig. 2 ▸ *b*). In the latter case, diffraction of the incident beam creates spatio-temporal echoes in the diffracted and transmitted beam represented by the blue dashed lines in Fig. 2 ▸(*c*), a phenomenon that is discussed in more detail in §2.2 for the transmitted beam.

It is important here to remark that amplitude and phase of the electric field in the transmitted beam are independent of the position of the crystal along the beam propagation direction. Indeed, the free-space propagator (5) and the through-crystal propagator (6) commute because they are both multiplicative in Fourier space. This implies that the X-ray intensity observed in the waist at 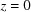 in Fig. 2 ▸(*c*) does not change as the crystal moves upstream from the waist into the convergent-beam region. A similar but more involved consideration applies to the diffracted beam, but is beyond the scope of this work. The total intensity of the diffracted beam is however independent of the crystal *z* position.

### 2.2. Spatio-temporal echoes   

An ideally collimated X-ray pulse propagating in a certain direction is represented by a vertical, infinitely thin oval in the DuMond diagram of Fig. 2 ▸(*a*). When such a short pulse propagates through a perfect thin crystal oriented to generate a diffracted beam, one observes beats of X-ray intensity that are delayed with respect to the main high-intensity pulse (Geloni *et al.*, 2010 ▸; Shvydko & Lindberg, 2012 ▸). These beats have been called temporal echoes. Their origin is explained qualitatively in a simple way by Geloni *et al.* (2010 ▸) as a consequence of the shape of the transmission coefficient 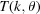 along the momentum coordinate *k* at fixed incident angle θ.

A monochromatic X-ray beam of narrow waist is represented by a horizontal, infinitely thin oval in the DuMond diagram of Fig. 2 ▸(*a*). When such a beam traverses the crystal, part of the X-ray intensity is displaced transversely and appears as lateral humps, called spatial echoes, in a near-field image at the beam waist (Bushuev, 2008 ▸; Bushuev & Samoylova, 2011 ▸; Bushuev & Oreshko, 2007 ▸). Their origin is explained in the same way as for the temporal echoes, because the behaviour of *T* along θ at fixed *k* is similar.

Shvydko and Lindberg recently pointed out that, in the general case, the incoming beam shows both a temporal and spatial structure, so that temporal and spatial echoes appear together (Lindberg & Shvydko, 2012 ▸; Shvydko & Lindberg, 2012 ▸). The time delay and the transverse displacement associated with an echo are related linearly by 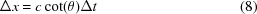
[see Figs. 6 and 9 of Shvydko & Lindberg (2012 ▸)].

In order to interpret the experimental data presented later, we simulated systematically the propagation of an X-ray pulse through a thin, infinitely extended perfect crystal, placed anywhere upstream of the beam waist.

The incoming pulses were defined in Fourier space as 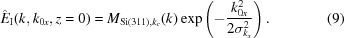


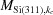
 is the transmission of a two-bounce symmetric Si(311) crystal monochromator set to the photon energy 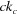, and represents the monochromator output of a perfectly collimated broadband pulse. The second factor implements the effect of an ideal, achromatic focusing element that makes the beam Gaussian and enforces the beam divergence 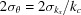. The waist is located at 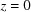 and the amplitude shows a Gaussian profile with an r.m.s. width of 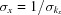. We performed simulations for the configurations that were measured experimentally. The width of the intensity profile of the beam at the waist was set to the FWHM value 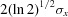 = 1.5 µm corresponding to the setup described in §3.2.

In the following, two concrete examples at the photon energy of 12 keV are discussed in more detail. The Darwin width of the Si(311) monochromator is 9.21 × 10^−6^, which gives an energy bandwidth of 0.32 eV. The intensity width at the waist of 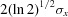 = 1.5 µm FWHM was enforced by setting the beam divergence to 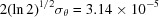 FWHM. The diamond (220) reflection was considered, which gives a Bragg angle of 24.1810° and a Darwin width of 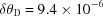.

In the first example, a diamond crystal of thickness 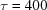
 µm was assumed in symmetric Bragg geometry (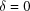). The reference *z* direction was set such that 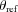 = 24.1810°, which gives the maximum of *R* at exactly 12 keV, and the simulations were performed for a number of beam energies 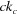 in a narrow range around 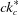 = 12 keV.

**Figure 3:**
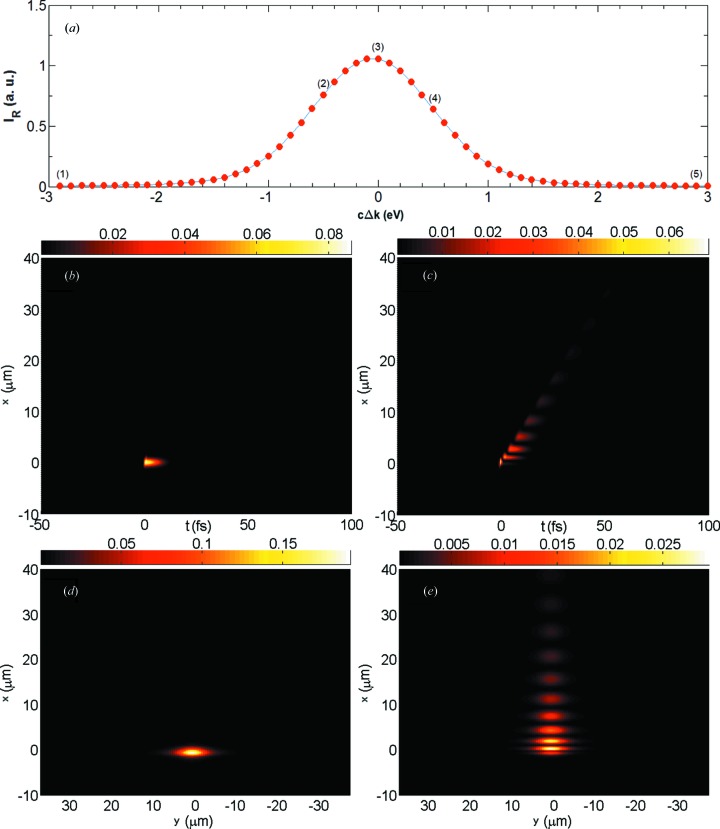
(*a*) Simulated reflectivity curve 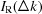 for the (220) reflection in symmetric Bragg geometry of a 400 µm-thick diamond single crystal at 12 keV. (*b*), (*c*) Simulated transverse intensity profile 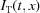 in the *x* direction as a function of time *t* of the transmitted beam at the beam waist downstream of the crystal: (*b*) photon energy 3 eV below the maximal diffraction condition, corresponding to point (1) in panel (*a*); (*c*) photon energy for the maximal diffraction condition, corresponding to point (3) in panel (*a*). (*d*), (*e*) Images of the intensity 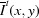 in the transverse 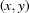 plane integrated over time for the configurations of panels (*b*) and (*c*), respectively.

Fig. 3 ▸(*a*) shows the reflectivity curve for the energy scan. The total intensity in the diffracted beam is calculated by the integration 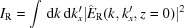 and is displayed as a function of the energy difference 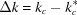. Figs. 3 ▸(*b*) and 3 ▸(*c*) show the time evolution of the intensity profile of the transmitted beam at the waist downstream of the crystal, calculated using equations (6) and (4), followed by the quadrature 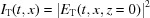. It is evident that the X-ray pulse is unaffected by the crystal in the off-Bragg case (*b*). In the on-diffraction condition case (*c*) we observe the intensity humps of the spatio-temporal echoes with the characteristic signature of equation (8). The 0th-order echo is neither delayed nor displaced, and can therefore be interpreted as the direct beam. Its intensity is much lower than that of the direct beam in the off-diffraction case, because photons are distributed over the other echoes and to the diffracted beam. These observations are perfectly in line with the results presented by Shvydko & Lindberg (2012 ▸), the difference being that here we use a monochromatic instead of a white incoming beam. In Figs. 3 ▸(*d*) and 3 ▸(*e*), we show, again for the off- and on-diffraction conditions, the intensity distribution 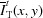 in transverse space after projection over time. For the sake of visual comparison with the experimental images presented later, we added the second transverse spatial dimension *y*. To the right-hand side of equation (9), we added the multiplicative Gaussian factor 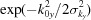 to give a spatial structure in *y*. The additional dimension is trivial, meaning that the *y* dependence of electric field and intensity is not altered by the crystal, which follows from the fact that the transmission and reflection coefficients are independent of the *y* coordinate.

In the second example, the diamond crystal had a thickness 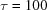
 µm and was oriented in symmetric Laue geometry (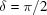). The reference *z* direction was again set such that the maximum of *R* was exactly at 12 keV, which for symmetric Laue geometry means 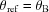 = 24.1810°. The simulations were performed analogously to the first example, and the equivalent results of Fig. 3 ▸ are shown in Fig. S1 in the supporting information.

### 2.3. Analysis of the echo signal   

**Figure 4:**
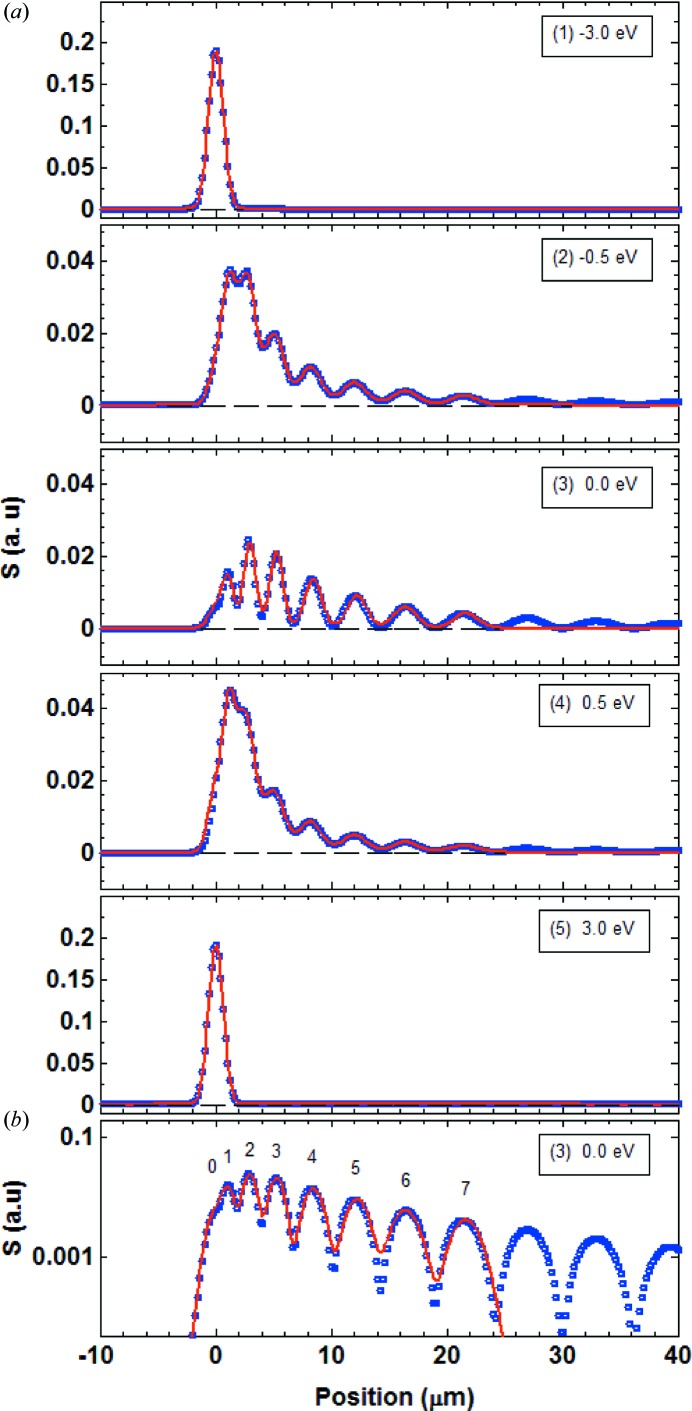
(*a*) Transverse echo signals 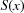 at the five points along the reflectivity curve indicated in Fig. 3 ▸(*a*). The energy deviation from the maximal diffraction condition is indicated. The blue dots are the data extracted from the simulated images [see examples in Figs. 3 ▸(*d*), (*e*)], while the red curves are the modelled multi-peak function [equation (11)]. (*b*) Same as (*a*) for the on-diffraction condition point (3) but in vertical logarithmic scale. The positions of the direct beam hump (0th-order echo) and of the transverse echoes are indicated.

We define the echo signal as the projection over time and over the trivial transverse space dimension, 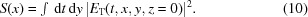
Fig. 4 ▸ shows the time-integrated echo signal, 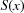, at the five different photon energies indicated on the reflectivity curve for the symmetric Bragg case example of Fig. 3 ▸(*a*). Away from the diffraction condition (points 1 and 5), only the unperturbed, direct-beam signal appears. Inside the on-diffraction condition window (points 2, 3 and 4), the echoes are observed as a series of maxima. Each signal at a given photon energy 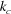 was modelled with a multiple Gaussian peak function of the form 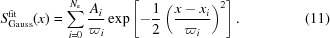


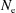
 is the number of echoes that are considered for the modelling. The parameters 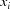, 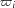 and 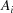 are the position, the r.m.s. width and the integrated signal of the *i*th echo, respectively. The full width at half-maximum is given by FWHM = 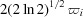. Outside the diffraction window, a single peak was sufficient to fit the model function to the signal, while for the data inside the diffraction window we used 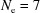 Gaussian peaks. The echo positions 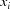 and widths 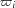 were determined by fitting at the maximal diffraction point (3), and were then kept unchanged for fitting at all other photon energies 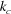. The values of all fitted parameters for the two representative energies 3 eV below diffraction condition (1) and at maximal diffraction (3) are reported in Table 1 ▸ (left). The obtained model functions are shown as red curves in Fig. 4 ▸ and reproduce well the echo signal data, as can be seen in particular in the logarithmic representation in Fig. 4 ▸(*b*).

**Figure 5:**
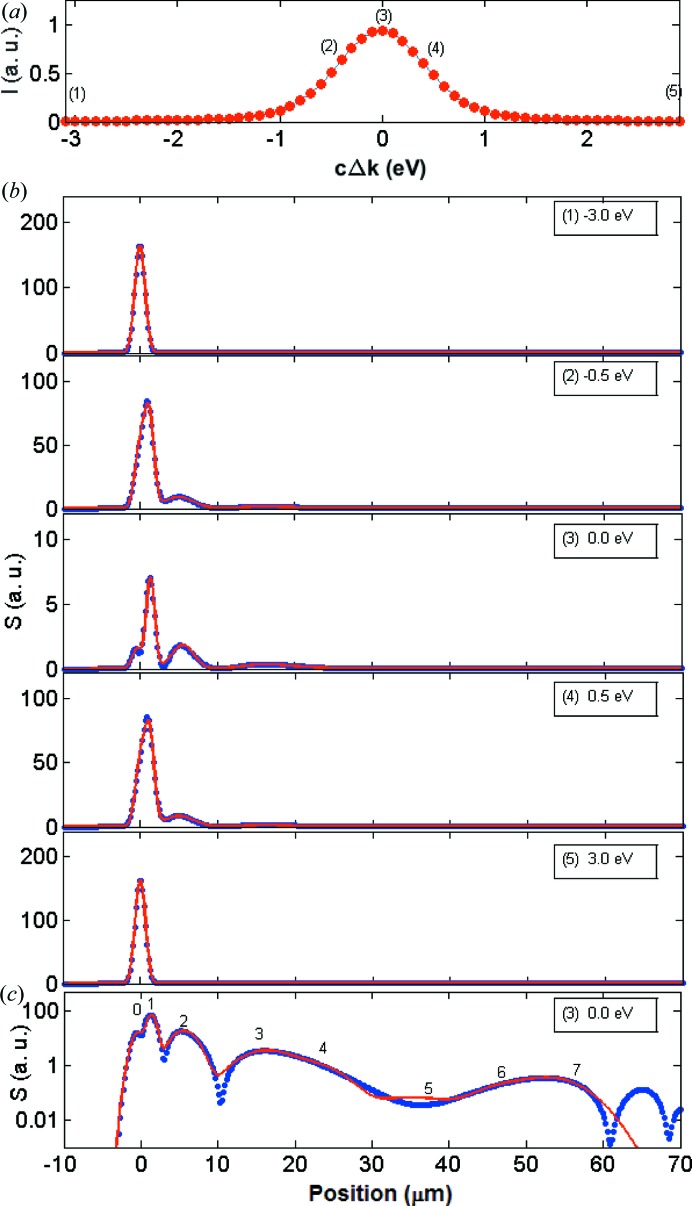
(*a*) Simulated reflectivity curve 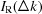 for the (220) reflection in symmetric Laue geometry of a 100 µm-thick diamond single crystal at 12 keV. (*b*) Transverse echo signals 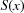 at the five points along the reflectivity curve shown in (*a*). The energy deviation from the maximum diffraction condition is indicated. The blue dots are the data extracted from the simulated images, while the red curves are the modelled Gaussian multi-peak function [equation (11)]. (*c*) Same as (*b*) for the on-diffraction condition point (3) but in vertical logarithmic scale. The positions of the direct-beam hump (0th-order echo) and of the transverse echoes are indicated.

Fig. 5 ▸ shows the reflectivity curve [panel (*a*)] and the echo signals with the corresponding modelling function [panels (*b*), (*c*)] for the Laue case example. The analysis of the echo signals was performed in the same way as for the Bragg case example, and the fitting parameters for the off- and on-diffraction conditions are listed in Table 2 ▸ (left). As shown in the logarithmic presentation in Fig. 5 ▸(*c*), the shape of the echo signal differs markedly from the symmetric Bragg case. The echo humps are not clearly visible, in particular those with displacements between 15 and 60 µm. Their approximate positions to be used as a starting value for fitting at the on-diffraction point (3) were inferred from a series of simulations with crystals with various thicknesses close to 100 µm (see Fig. S2 in the supporting information).

As a final remark concerning the simulations, it should be pointed out that the spectra and temporal shapes assumed for the incoming X-ray pulses are not realistic for synchrotron or SASE-FEL X-ray sources. The real spectra exhibit narrow spikes of width corresponding to the inverse pulse length, of the order of 50 ps and 10 fs, respectively. Similarly, the internal time structure of these pulses contains spikes of duration equal to the inverse bandwidth. To reproduce for example the experiments reported in this work with 50 ps synchrotron pulses, both the direct beam and the echoes of Figs. 3 ▸(*b*), 3 ▸(*c*) would have to be convoluted with the suitable time structure. However, this operation does not affect the time-integrated intensity, and therefore neither does it affect the echo signals.

## 3. Experiment   

### 3.1. Crystal samples   

The samples under study were three diamond single crystals of 100, 400 and 500 µm thickness, with a square shape of 5 mm edge length. From Laue diffraction studies performed with a laboratory X-ray source, we established the surface normal to be parallel to the (110) reflection direction for the 400 µm-thick crystal, and parallel to the (100) reflection direction for the 100 and 500 µm-thick crystals. For later convenience, we label the three crystals as C_100µm_ (100), C_400µm_ (110) and C_500µm_ (100).

### 3.2. Experimental setup   

The aim of the experiment was to observe and characterize the echoes at the beam waist produced by the thin diamond crystals. The measurements were performed at the microXAS beamline of the Swiss Light Source, which satisfies the three key requirements of monochromaticity, photon-energy range and focus size to succeed.

**Figure 6:**
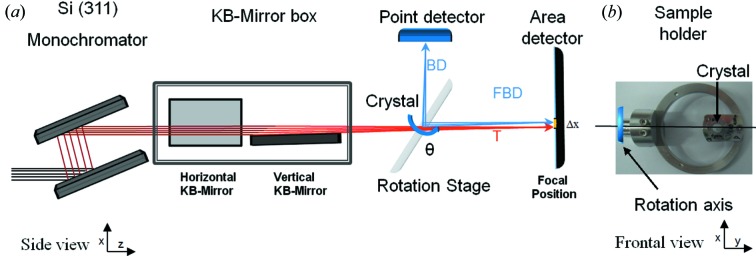
(*a*) Schematic representation of the experimental setup implemented at the microXAS beamline. (*b*) Picture of sample C_400µm_ (110) mounted in the sample holder. The rotation axis is in the *y* direction.

The experimental configuration is sketched in Fig. 6 ▸(*a*). The perfectly collimated incoming beam was first sent through the microXAS Si(311) two-bounce monochromator, which sets the relative bandwidth to values of the order of the Darwin width of the diamond reflections under study. The beam was then focused vertically and horizontally by two bendable Kirkpatrick–Baez (KB) mirrors to a spot size of 1.5 (v) × 10 (h) µm FWHM at 100 mm distance downstream of the mirror box exit window. The vertical focus size was determined by scanning a sharp edge in the vertical direction and evaluating the sharpness of the step of the transmitted intensity in the forward direction. The vertical divergence of the focused beam was reduced as much as possible by closing the vertical slits upstream of the KB mirror box until just before the focused beam spot is disturbed markedly. This reduces the area in the DuMond diagram not involved in diffraction. From the slit aperture of about 30 µm and the distance between the vertically focusing KB mirror and the focus of 280 mm, the divergence of the incoming beam is estimated to be about 10^−4^, *i.e.* about a factor of 3 times larger than in the Gaussian beams with the same focus size used in the simulations.

An individual diamond single crystal was mounted on a rotation stage with the horizontal rotation axis intersecting the sample and perpendicular to the beam direction. An example crystal mount is shown in Fig. 6 ▸(*b*). The rotation angle could be controlled with a precision of 0.0005°. The whole rotation stage could be moved vertically and horizontally in order to get the beam onto the diamond, and along the beam direction to place the sample about 60 mm upstream of the focal point. At the focal position, a crystal scintillator was placed with the surface perpendicular to the beam direction and imaged with 20-fold magnification onto a pco.2000 camera with pixels of 7.4 µm size, giving thus a theoretical resolution of 0.37 µm. The intensity of the beam diffracted by the diamond was measured using a diode as point detector, which could be moved in the vertical diffraction plane to a suitable position to intercept the diffracted beam.

### 3.3. Data collection   

For the measurements, the photon energy was first set with the monochromator to a reference value and an angular rocking scan [red path in Fig. 2 ▸(*a*)] was performed to determine the incidence angle at which the sample was in the diffraction condition. The reflectivity curve of these rocking scans was collected using the point detector located at the expected Bragg angle, as shown in Fig. 6 ▸(*a*). Implicit in this procedure is that we targeted only reflections with vertical diffraction geometry with σ polarization. Once the exact angle for the reference photon energy was set, an energy scan [cyan path in Fig. 2 ▸(*a*)] with steps of 0.5 eV over an energy range of 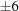
 eV was performed. For each energy point in the scan, ten subsequent images, each of 0.1 s exposure, were recorded with the forward area detector to avoid saturation. The ten images were then added up to obtain a single image. For each energy step, the diffracted beam intensity was measured simultaneously with the point detector. Systematic measurements at 10 and 12 keV were attempted with each crystal for the lowest-order reflection parallel to the surface normal (symmetric Bragg geometry), and for the lowest-order reflection parallel to the surface normal and to the crystal edges (symmetric Laue geometry).

## 4. Results   

**Figure 7:**
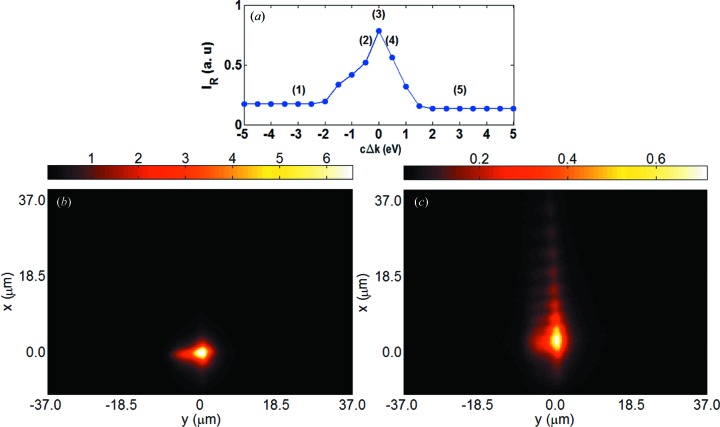
(*a*) Experimental reflectivity curve 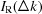 collected for the (220) reflection at 12 keV in symmetric Bragg geometry with the 400 µm-thick diamond crystal. (*b*), (*c*) Intensity images collected with the detector placed at the vertical focus position of the incident beam for (*b*) a photon energy of 3 eV below the perfect Bragg condition, corresponding to point (1) in panel (*a*), and for (*c*) a photon energy centred perfectly at the Bragg condition, corresponding to point (3) in panel (*a*).

Fig. 7 ▸ shows results obtained from an energy scan at 12 keV with the C_400µm_ (110) crystal; the reflection presented is the (220) reflection in symmetric Bragg geometry, which corresponds to the first simulation described in §2.2. Panel (*a*) shows the measured reflectivity curve 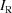, which allows discrimination between the off- and on-diffraction conditions. Panels (*b*) and (*c*) display the image of the transmitted beam for the off- and on-diffraction conditions, respectively. The intensity humps in the vertical direction appear only in the latter case, in agreement with the simulation results shown in Figs. 3 ▸(*d*) and (*e*).

**Figure 8:**
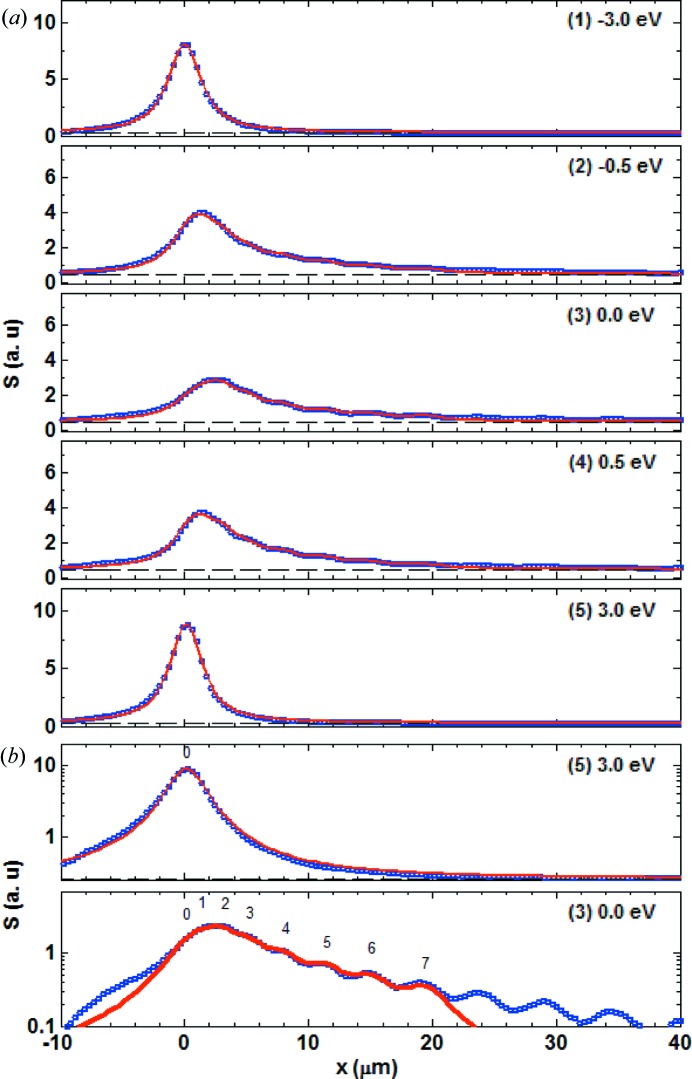
(*a*) Transverse echo signals 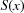 at the five points along the reflectivity curve of Fig. 7 ▸(*a*). The energy deviation from the maximal diffraction condition is indicated. The blue dots are the data extracted from the experimental images [see examples in Figs. 7 ▸(*b*), (*c*)], while the red curves are the modelled Lorentz multi-peak function [equation (12)]. (*b*) Same as (*a*) for the off-Bragg condition point (1) and for the on-Bragg condition point (3) but in vertical logarithmic scale. The position of the direct-beam hump (0th-order echo) and of the transverse echoes is indicated.

Fig. 8 ▸ displays the experimental echo signals 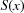 for five energy scan points, calculated by horizontal *y* projection of the transmitted beam images in a 10 µm-wide strip onto the vertical axis *x*. These signals are the analogues of the intensity projections obtained with equation (10) from the simulated transmitted beam images.

Again, in analogy to the fitting procedure for the simulated data based on equation (11), the echo signals were modelled with the function 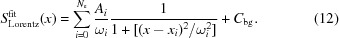
Instead of Gaussians, Lorentzian peak shapes were used to account for the broader peak base, observed even without a crystal placed in the beam. A constant 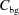 was added to account for the overall background in the experimental images. The variations of 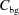 between different energies were less than 1% of the amplitude of the signal at the off-diffraction condition and are therefore irrelevant.

For the off-diffraction condition points (1) and (5), the echo could be fitted with a single peak (0th-order echo) at fixed position 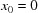
 µm and FWHM 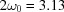
 µm, which exceeds the expected value of 1.5 µm from the knife-edge scan by about 1.6 µm. We attribute this discrepancy to broadening originating from the thickness of the scintillator crystal and possible aberrations in the optical elements of the camera. The agreement between the data and the fits is good, except for the weaker side lobes shown in Fig. 8 ▸(*b*) for the energy point (5). These lobes are most likely to result from diffraction from the beam-defining slits upstream of the focusing mirrors and cannot be modelled easily.

To model the echo signal for the on-Bragg energy points (2–4), seven additional echoes (*i* = 1–7) were considered in equation (12) and a protocol similar to that used for the simulation was applied. The modelled functions are shown as red curves in Fig. 8 ▸ and reproduce reasonably well the experimental data points, as is shown in particular in the logarithmic presentation of Fig. 8 ▸(*b*) for the energy point (3). The fitting parameters for the off-diffraction energy point (1) and for the on-diffraction energy point (3) are reported in Table 1 ▸ (right). The echo displacements agree extremely well with the values predicted by the simulations, while the FWHMs appear to be larger by 1.6 to 2.0 µm. The constant part of this broadening is attributed, as for the off-Bragg point, to the imperfection of the imaging system. The remaining part of the broadening, which is most pronounced at the lowest echo orders, is most likely due to the fact that the real X-ray beam is far from being an ideal Gaussian beam. This means that the phases of the Fourier space components of the incoming and transmitted beams in equation (6) are scrambled, which typically results in broadening of the real-space intensity pattern at the beam waist.

**Figure 9:**
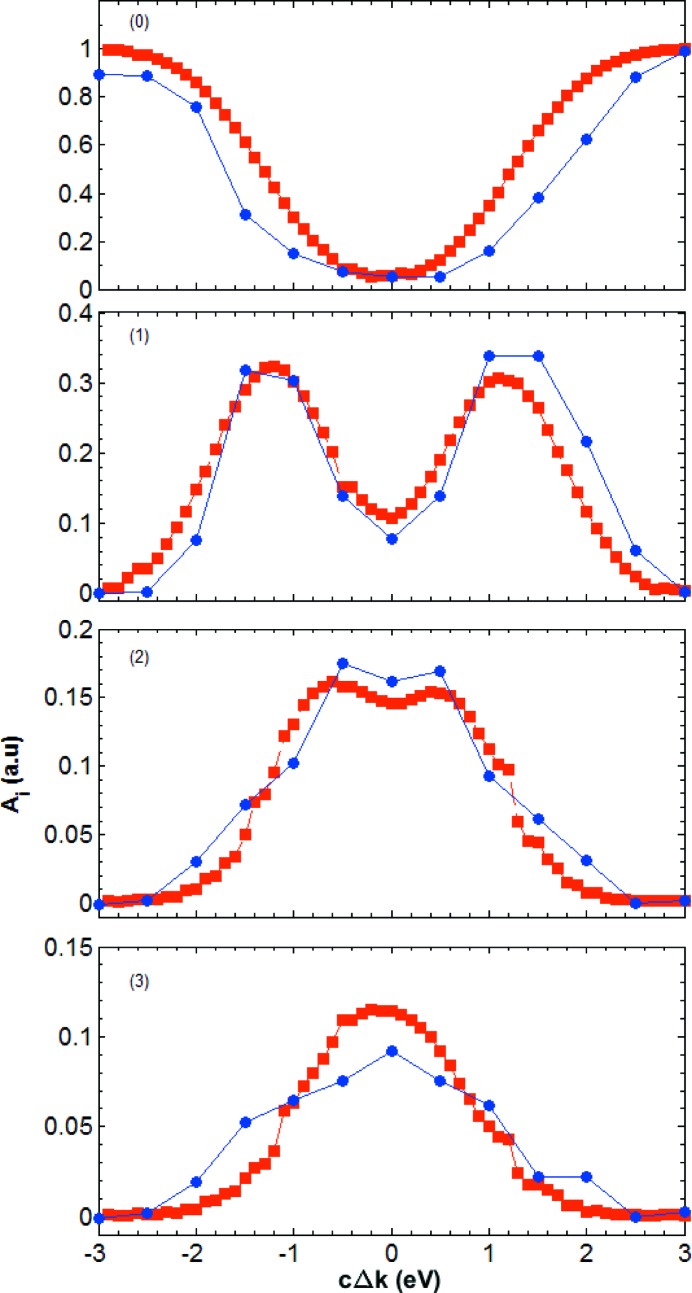
Energy dependence of the integrated echo intensity corresponding to the data of Figs. 7 ▸ and 8 ▸. The blue circles are the values obtained from the experimental data, while the red squares are from the simulations. The intensities 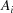 of the direct beam (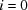) and of the first three echoes (*i* = 1–3) are obtained by modelling the data with the multi-peak functions [equations (11) and (12)], and are shown as a function of the energy difference 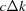 with respect to the maximal diffraction condition.

Fig. 9 ▸ compares the experimental and simulated intensities of the direct beam (0th-order echo) and the first three transverse echoes as a function of photon energy. The overall trend of both signals is the same. The transmitted beam intensity is minimal for maximal diffraction (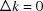). The intensity of the echoes has a symmetric two-hump profile. The distance between the humps decreases with increasing order *i* of the echo. For example, for 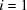 the hump separation is about 3.0 eV, for 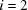 it is about 1.6 eV, while for 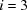 the two humps are not visible anymore.

**Figure 10:**
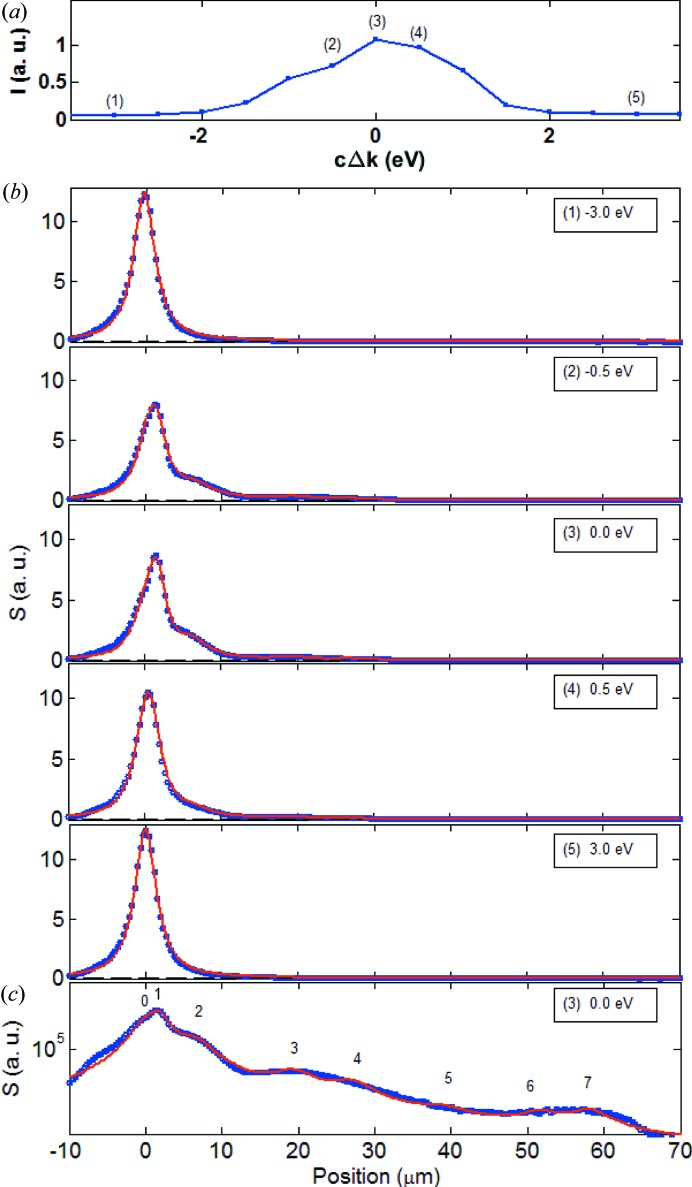
Same as Fig. 8 ▸ but for the (022) reflection in symmetric Laue geometry of a 100 µm-thick diamond crystal at 12 keV.

Figs. 10 ▸ and 11 ▸ as well as Table 2 ▸ (right) are the equivalent of Figs. 8 ▸ and 9 ▸ and Table 1 ▸ (right) for the measurements performed with the C_100µm_ (100) crystal on the (022) reflection in symmetric Laue geometry. This corresponds to the second simulation described in §2.2. An example of transmitted beam images is shown in Fig. S3 in the supporting information. The data acquisition and analysis procedure was the same as for the Bragg geometry case above. Because the humps are hard to identify in the experimental echo signals but the overall signals are similar to those from the simulations (Fig. 10 ▸ *c*), the modelling was done with echoes placed at the same positions as in the simulations before fitting. Overall, the modelling of the experimental and simulated echo signals gives a consistent outcome, even though the agreement is less remarkable if compared to the Bragg case.

**Figure 12:**
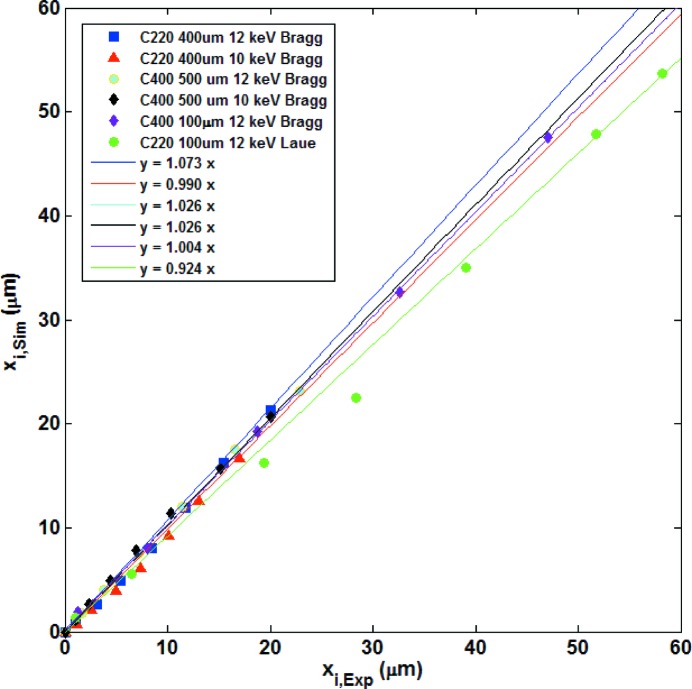
Comparison of transverse echo displacements from experiment and simulations. Each data point is related to the displacement 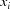 of a certain echo at the maximum diffraction condition determined from the experimental data (horizontal axis) and from the simulations (vertical axis). Each colour is for a specific crystal, photon energy and geometry, indicated in the plots. The lines represent the best linear correlation and follow the same colour coding. The slope values are given in the legend.

In all, during the beamtime we attempted to collect data in both Bragg and Laue symmetric geometries at 10 and 12 keV on the two thicker crystals C_400µm_ (110) and C_500µm_ (100), and at 12 keV only on the thin crystal C_100µm_ (100). In the Bragg case, all five measurements were successful, where success means that at least the first two displaced echo peaks could be clearly identified and reasonably modelled with equation (12). In more detail, the measurements were successful with the C_400µm_ (110) crystal on the (220) reflection at 12 keV (see the data presented above) and 10 keV (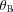 = 29.44°), with the C_500µm_ (100) crystal on the (400) reflection at 12 and 10 keV (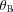 = 35.40° and 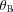 = 44.04°, respectively), and with the C_100µm_ (100) crystal at 12 keV on the reflection (400). In the Laue case, only one of the five measurements was successful, namely that with the thinner C_100µm_ (100) crystal at 12 keV on the (022) reflection (see the data presented above). The exhaustive comparison of the peak positions derived from the simulated and experimental echo signals is the subject of Fig. 12 ▸, which demonstrates that for all measured reflections the corresponding echo positions are well correlated. The correlation is particularly good for the C_100µm_ (100) and C_500µm_ (100) sample in the Bragg case. Regarding the Laue case, we remark that setting the crystal in the right orientation for diffraction is much more challenging than in the Bragg case, because of the additional degree of freedom represented by in-plane rotations of the crystals which was hard to control. This explains both the fact that most attempts in Laue geometry were unsuccessful, because of the difficulty of properly orienting the crystal in the diffraction condition, and the fact that in the sole successful Laue measurement the echo-position correlation is worse than that in Bragg geometry, given that even a small asymmetry has a remarkable effect on the echo position (simulations not shown here).

## 5. Discussion   

Experiments on FBD by a thin crystal were performed decades ago to study the *Pendellösung* effect [in Bragg gemetry by Kato & Lang (1959 ▸) and in Laue geometry by Batterman & Hildebrandt (1968 ▸)], which consists of oscillations of the transmitted-beam intensity upon small variations of the incidence angle or wavelength of the incoming plane wave. These are far-field experiments, for which the X-ray intensity is detected as a function of the propagation angle, *i.e.* as 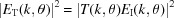. Analogous studies were also done on the diffracted beam (Mocella *et al.*, 2000 ▸). In contrast, the generation by FBD of echoes is a near-field phenomenon, and involves interference of plane waves of different photon energies and/or different incidence angles. As mentioned in §1, this phenomenon has been investigated deeply from the theoretical point of view in recent years, in relation to the X-ray self-seeding possibilities at XFEL facilities. Indirect experimental evidence for the retardation of temporal echoes is given by the fact that self-seeding at an XFEL has been implemented, while for the transverse spatial displacement the hints come from the fact that the trajectory of the electron beam in the downstream undulator section has to be adjusted correspondingly.

The results reported in this article represent the first direct evidence of the echoes. The success of the experiment relies on two key points. First, at the microXAS beamline it was possible to achieve a focus size of 1–2 µm, which is suitable for direct visualization of the echoes at the focal plane of the incident beam. In parallel, by using microXAS it was also possible to minimize the incident-beam divergence and therefore minimize the fraction of photons not involved in the diffraction process that are transmitted into the 0th-order echo hump. Second, because the longitudinal position of the crystal upstream of the imaging plane is not critical, it was possible to physically separate the position of the area detector, set at the focal plane, from the position of the crystal, mounted on the bulky rotation stage placed upstream of the focus. To support this concept, we recorded images of the transmitted beam at focus with the crystal set to the Bragg condition at three distances, differing by 10 mm, upstream of the focus, and established that the three images were practically the same, showing identical position and similar intensities for each of the echoes in the different crystal locations (see Fig. S4 in the supporting information).

The echo signals extracted from the experimental images measured at the focal position are in good agreement with those generated using the simulation tool, leaving no doubts as to the fact that real echoes were effectively observed and providing indirect evidence that equation (8) is fulfilled in practice. Minor quantitative discrepancies are attributed to the difficulties in modelling the details of the experimental beam shape. The simulation tool was developed with the precise aim of handling an incident X-ray pulse of chosen temporal and spatial shape, and will serve for future studies related to self-seeding.

Direct visualization of the echoes in the time domain cannot be done at a synchrotron source, because it requires femtosecond resolution. The combination of tight focusing with a Si_3_N_4_ screen placed at the focus, in the configuration of a timing tool as presented by Harmand *et al.* (2013 ▸), and with the time dimension orthogonal to the echo displacement direction could make it possible to determine the fine delay of the echoes. This experimental arrangement would also be suited to exploiting the strict correlation of the time delay and spatial displacement of the echoes to study the dynamics of strain relaxation in thin crystals of silicon, as induced by short laser pulses.

Since the shape of the echo signal is very characteristic for a given crystal thickness and reflection, one could consider exploiting the echoes as an online diagnostics tool in a self-seeding module based on the design of Geloni *et al.* (2010 ▸). For practical implementation, two problems have nevertheless to be addressed. First, most of the SASE beam does not contribute to the echoes, but rather its high intensity is an obstacle to proper identification and quantification of the echo signal. Second, the transversal displacement of the echoes of the order of a few µm means the incoming beam needs to be focused to a size which is of the same order. An XFEL beam waist is typically much larger. A possibility would be to insert a grating of a few µm upstream of the seeding crystal to generate a weak secondary beam with small horizontal deflection, such that it hits the seeding crystal with almost the same direction as the direct beam. This secondary beam would then be refocused onto a screen to make the echoes visible. Adding a monochromator crystal to the secondary beam would in addition suppress the direct beam on the screen.

## 6. Summary   

We have reported the first experimental observation of transversely displaced echoes generated *via* forward Bragg diffraction of an X-ray beam propagating through a perfect thin crystal. The agreement of the experimental echo signal with that obtained from simulations relying on the dynamical diffraction theory is very good. This paves the way for the imaging of the echoes as a tool to diagnose forward-diffracted beams as applied in self-seeding modules or to study temporal strain effects in thin perfect crystals.

## Supplementary Material

Additional figures to illustrate some assumptions made during analysis of the simulated and experimental data. DOI: 10.1107/S2053273318001419/sc5112sup1.pdf

## Figures and Tables

**Figure 11:**
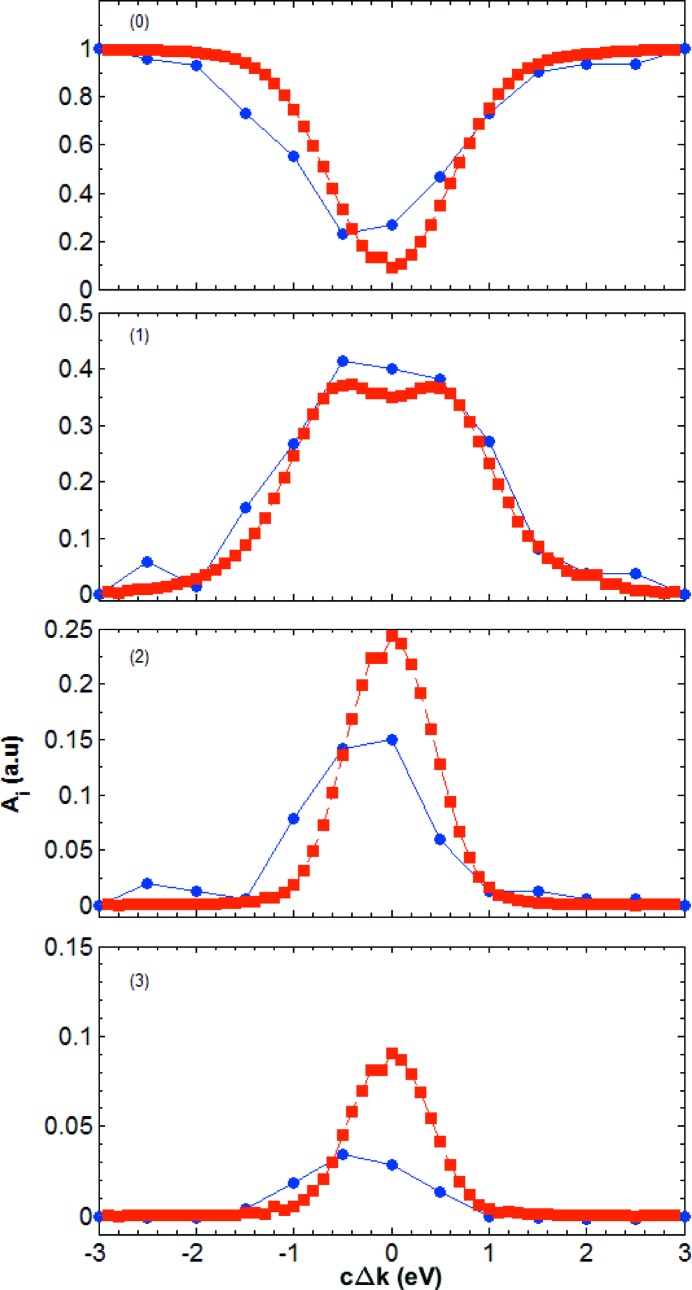
Same as Fig. 9 ▸ but for the (022) reflection in symmetric Laue geometry of a 100 µm-thick diamond crystal at 12 keV.

**Table 1:** Fitting parameters from a 400 µm-thick crystal for the (220) reflection at 12 keV in symmetric Bragg geometry for (left) modelling the simulated echo signals 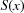 with the Gaussian multi-peak function [equation (11)] and for (right) experimental echo signals 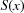 with the Lorentz multi-peak function [equation (12)] The values are reported for an energy of 3 eV below the perfect Bragg condition, for which a single peak was sufficient, and for the perfect Bragg condition, for which seven additional echo peaks were modelled. The reported parameters are the energy deviation 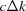, the order of the echoes *i*, the transverse echo displacement 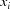, the full width at half-maximum of the echoes FWHM_*i*_, the integrated intensity of the echoes 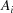 and the 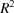 value of the fitting procedure.

		Simulation				Experiment			
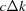 (eV)	*i*	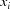 (µm)	FWHM (µm)	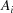 (a.u.)	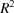	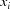 (µm)	FWHM (µm)	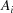 (a.u.)	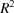
−3.0	0	0.00	1.51	1.00	0.99997	0.00	3.23	1.00	0.99997
									
0.0	0	0.0	1.51	0.04	0.96	0.00	3.23	0.04	0.98
	1	1.1	1.15	0.10	—	1.05	3.13	0.08	—
	2	2.9	1.24	0.15	—	3.15	3.46	0.15	—
	3	5.3	1.32	0.11	—	5.43	3.57	0.10	—
	4	8.4	1.75	0.08	—	8.45	3.62	0.06	—
	5	12.2	1.99	0.06	—	11.79	4.03	0.04	—
	6	16.4	2.41	0.04	—	15.49	4.04	0.04	—
	7	21.6	2.49	0.03	—	20	4.05	0.03	—

**Table 2:** Fitting parameters from a 100 µm-thick crystal for the (022) symmetric Laue geometry reflection at 12 keV for (left) modelling the simulated echo signals 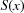 with the Gaussian multi-peak function [equation (11)] and (right) experimental echo signals 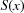 with the Lorentz multi-peak function [equation (12)] The values are reported for an energy of 3 eV below thte perfect Bragg condition, for which a single peak was sufficient, and for the perfect Bragg condition, for which seven additional echo peaks were modelled. The reported parameters are the energy deviation 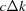, the order of the echoes *i*, the transverse echo displacement 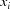, the full width at half-maximum of the echoes FWHM_*i*_, the integrated intensity of the echoes 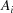 and the 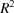 value of the fitting procedure.

		Simulation				Experiment			
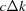 (eV)	*i*	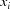 (µm)	FWHM (µm)	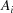 (a.u.)	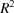	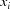 (µm)	FWHM (µm)	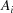 (a.u.)	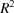
−3.0	0	0.00	1.50	1.00	1	0.00	3.35	1.00	0.999
									
0.0	0	0.00	1.50	0.100	0.98	0.00	3.35	0.285	0.98
	1	1.33	1.23	0.346	—	1.04	3.32	0.427	—
	2	5.51	3.32	0.243	—	6.47	6.13	0.161	—
	3	16.24	6.40	0.086	—	19.37	6.37	0.032	—
	4	22.54	8.43	0.030	—	28.34	5.60	0.015	—
	5	35.03	10.58	0.002	—	39.10	5.67	0.001	—
	6	47.89	8.86	0.006	—	51.77	5.57	0.002	—
	7	53.67	7.42	0.008	—	58.16	5.59	0.003	—
