# Supplementary material for: Spatial displacement of forward-diffracted X-ray beams by perfect crystals
Source: Acta Crystallogr A Found Adv. 2018 Feb 23;74(Pt 2):75–87. doi: 10.1107/S2053273318001419 (PMC5831586; doi:10.1107/S2053273318001419)
Supplement: Supplementary file 1 [file a-74-00075-sup1.pdf]

FOUNDATIONS ADVANCES

Volume 74 (2018)

Supporting information for article:

Spatial displacement of forward diffracted X-ray beams by perfect crystals

A. Rodriguez-Fernandez, V. Esposito, D. F. Sanchez, K. D. Finkelstein, P. Juranic, U. Staub, D. Grolimund, S. Reiche and B. Pedrini

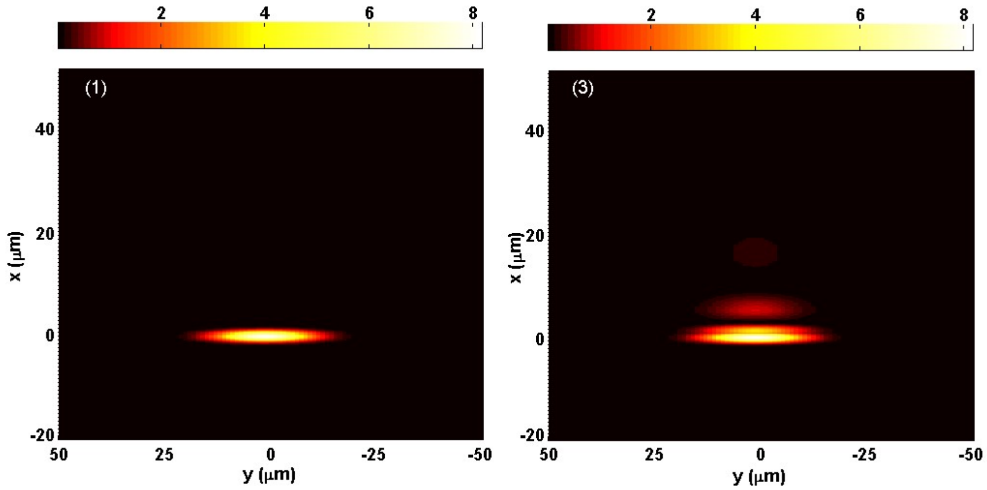

Fig. S1. Simulated transverse intensity profile $I_T(x,y)$ of the transmitted beam for a (022) reflection in Laue geometry at 12 keV at the beam waist downstream the crystal: (1) photon energy 3eV below maximal diffraction condition, corresponding to point (1) in panel Figure 5 (a); (3) photon energy for maximal diffraction condition, corresponding to point (3) in panel (a).

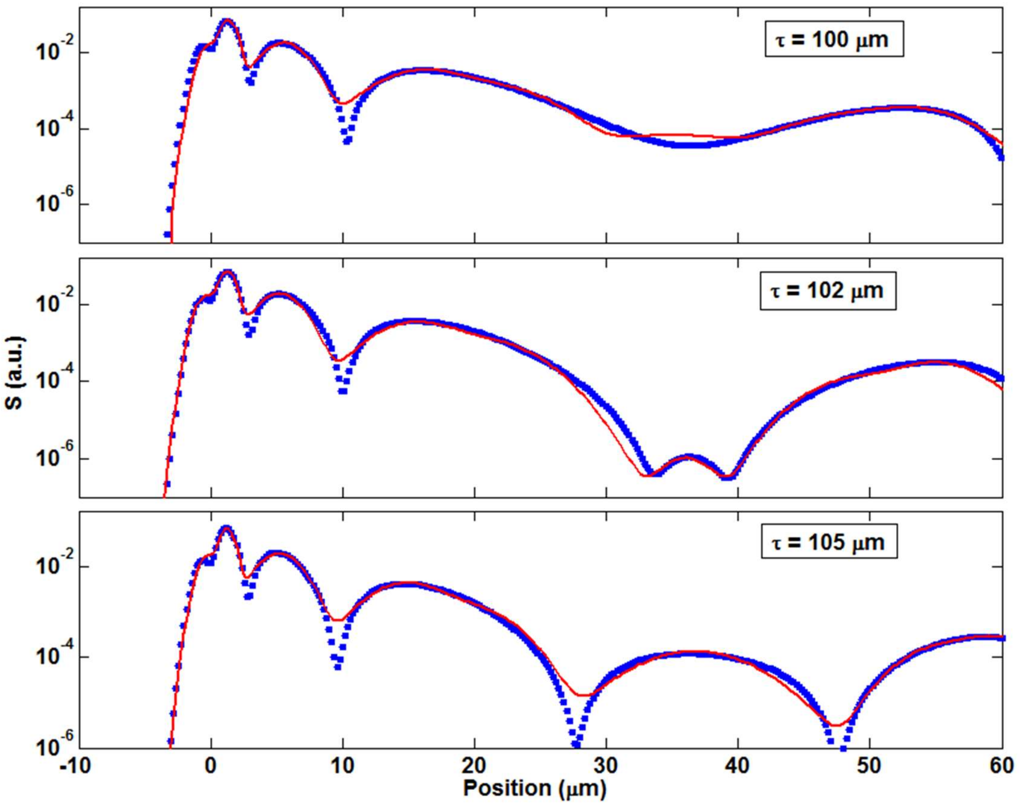

Fig. S2. Simulated transverse echo signals S(x) for the (022) reflection at 12 keV, three different thicknesses are presented 100, 102 and 105 μm. The blue dots are the data extracted from the simulated images, while the red curves are the modeled Gaussian multi-peak function 11. It is possible to observe the generation of different peaks in function of the thickness. This was used to help the fitting of the data.

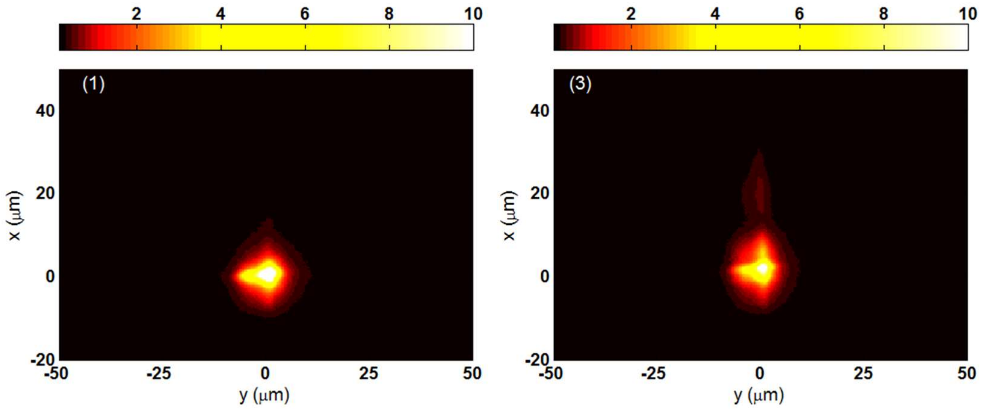

Fig. S3. Intensity images collected for the Laue (022) reflection at 12 keV with the detector placed at the vertical focus position of the incident beam for (1) a photon energy of 3 eV below perfect Bragg condition, corresponding to point (1) in Figure 10 (a), and for (3) a photon energy centered perfectly at Bragg condition, corresponding to point (3).

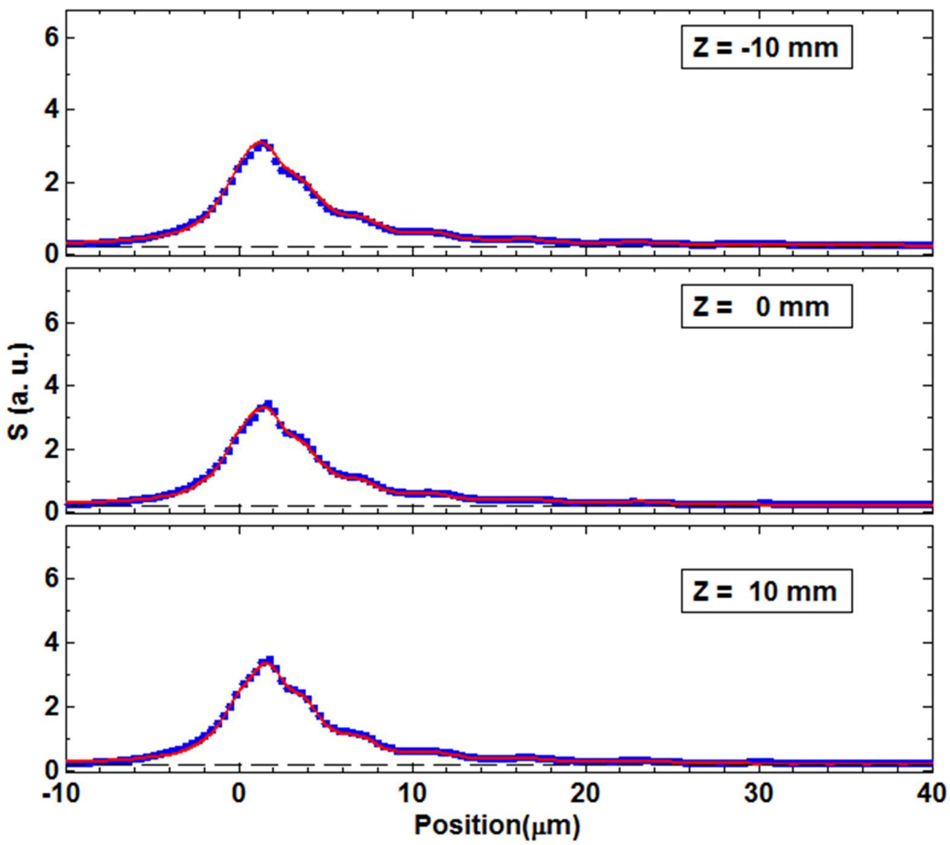

Fig. S4. Transverse echo signals S(x) for three different location of the diamond samples, each 10 mm away in the beam propagation direction. The reflection shown is the (400) reflection in the $C_{500\mu m}(100)$ collected at 13.2 keV. The blue dots are the data extracted from the simulated images, while the red curves are the modeled Gaussian multi-peak function 12.
